# Antibiofilm Potential and Mechanisms of *Lacticaseibacillus paracasei* L475 Against Multidrug-Resistant *Escherichia coli* Isolated from Older Adults

**DOI:** 10.3390/microorganisms14040888

**Published:** 2026-04-16

**Authors:** Botong Zhang, Sainan Guo, Mingyu Li, Yuan Niu, Yiman Liu, Nan Wu, Hong Zhu, Yue Cui

**Affiliations:** College of Food Science and Biology, Hebei University of Science and Technology, Shijiazhuang 050018, China; 13081035320@163.com (B.Z.); snguo0723@163.com (S.G.); 15612585921@163.com (M.L.); 18031381461@163.com (Y.N.); 13191678986@163.com (Y.L.); nndwunan@163.com (N.W.)

**Keywords:** *Lactobacillus*, antibiofilm, *Escherichia coli*, older individuals, multidrug-resistance

## Abstract

The high prevalence of biofilm-associated multidrug-resistant (MDR) *Escherichia coli* infections in older adults calls for novel control strategies. This study compared fecal *E. coli* carriage, antimicrobial resistance, and biofilm formation among community-dwelling older adults with different self-reported immune statuses (lower vs. normal), and evaluated the antibiofilm activity of five *Lactobacillus* cell-free supernatants (CFSs). Fecal samples from 20 older adults were analyzed. *E. coli* was enumerated, and isolates were characterized for antimicrobial susceptibility and biofilm formation. Five *Lactobacillus* strains were screened for antibiofilm activity using crystal violet assay, with further evaluation of extracellular polymeric substance (EPS) production and biofilm morphology. After removing the redundant isolates, 70 isolates were reported, with significantly higher counts in the lower-immunity group (7.89 vs. 6.04 log MPN/g). The lower-immunity group had significantly higher antimicrobial resistance (97.3% vs. 60.6%), and higher MDR prevalence (91.7% vs. 24.2%). Biofilm formation was observed in 62.9% of isolates, with significantly higher prevalence among MDR isolates and in the lower-immunity group. *L. paracasei* L475 CFS showed the strongest antibiofilm activity against a representative MDR isolate (L5-1), with inhibition and eradication rates of 82.9% and 75.0%, respectively. Mechanistically, L475 CFS reduced extracellular polymeric substance components, with a 92.3% reduction in proteins and 41.3% in polysaccharides. Microscopy confirmed disrupted biofilm architecture, membrane damage, and cell lysis. In conclusion, these preliminary findings indicate a potential association between self-reported immune function and *E. coli* resistance/biofilm formation in older adults. *L. paracasei* L475 CFS demonstrates promising in vitro antibiofilm activity against an MDR *E. coli* isolate from this population, supporting its potential as a postbiotic candidate.

## 1. Introduction

*Escherichia coli* is a diverse Gram-negative species of bacteria found in the human gut, where it can exist as a commensal, probiotic, or pathogenic microbe [1]. Although most *E. coli* strains are harmless to humans, some pathotypes can cause a wide range of infections in the gastrointestinal tract and other parts of the body, including the brain, urinary tract, and bloodstream, leading to conditions such as meningitis, urinary tract infections, and sepsis [2]. Previous studies have revealed higher *E. coli* colonization rates in older adults than in younger individuals [3], particularly among those with sepsis, inflammatory bowel disease, and urinary tract infections [4,5,6]. These findings underscore the clinical significance of *E. coli* in the health of older populations. Current treatment approaches for *E. coli* infections primarily involve antibiotic treatment. However, the rapid emergence of antimicrobial resistance, especially multidrug resistance (MDR), poses a global threat to public health. Older adults are particularly susceptible to these infections due to age-related immune dysfunction and the high prevalence of comorbidities [7]. Notably, *E. coli* has been identified as one of the most common commensal microorganisms exhibiting high MDR rates in both clinical and community settings [8,9]. As a result, MDR in *E. coli* is widely recognized as a major public health concern worldwide.

Biofilms are consortia of microorganisms surrounded by a self-produced extracellular polymeric matrix consisting of polysaccharides, proteins, and extracellular DNA. This matrix protects the embedded bacteria against host defenses and antimicrobial agents [10]. *E*. *coli* is a well-known biofilm-forming microorganism capable of colonizing intestinal surfaces, medical devices, and food products [11]. It is estimated that approximately 80% of chronic inflammatory and infectious diseases in humans are associated with bacterial biofilm formation, with nearly one million deaths annually attributed to such biofilm-related infections worldwide [12]. Furthermore, studies have reported that the prevalence of biofilm-associated MDR ranges from 17.9% to 100.0%, with *E. coli* isolates often showing the highest rates. This highlights the substantial burden of MDR via biofilm formation [13].

Biofilm formation, along with the high prevalence of *E. coli*-associated infections and the increasing emergence of MDR strains, has necessitated the development of novel strategies for biofilm control. It is well established that probiotics, particularly members of the genus *Lactobacillus*, could serve as promising agents for combating biofilm-associated infections. Probiotics are living microorganisms that confer health benefits to the host by enhancing the intestinal microbial balance, and *Lactobacillus* species are the predominant group of known probiotics. These bacteria are generally recognized as safe and have been widely explored for various therapeutic applications. Previous studies have demonstrated that *Lactobacillus* strains and their cell-free supernatants (CFSs) can effectively inhibit the biofilms of various pathogenic bacteria, either by preventing biofilm formation or by eradicating mature biofilms [14,15]. These antibiofilm effects have been attributed to multiple mechanisms, including alterations in bacterial cell surface morphology, the suppression of extracellular polymeric substance (EPS) production, and the modulation of biofilm-related genes [16]. However, most existing studies have focused on foodborne pathogens or laboratory model strains, with reported antibiofilm activities varying considerably across *Lactobacillus* species and strains [17]. Moreover, there is limited information on the antibiofilm efficacy of *Lactobacillus* CFSs against clinical *E. coli* isolates that are both strongly biofilm-forming and multidrug-resistant, particularly when isolated from older adults with self-reported lower immune status—a population more vulnerable to infection.

Therefore, the present study aimed to determine the antibiofilm effects of CFSs from several *Lactobacillus* strains against MDR *E. coli* isolates with strong biofilm-forming capacity obtained from older adults. Eventually, *Lactobacillus paracasei* L475 was selected for further investigation of its influence on EPS production and cell membrane morphology in *E. coli* biofilms. By focusing specifically on isolates from this vulnerable population, these analyses provide a theoretical basis and technical support for the development of effective strategies to control *E. coli* biofilm-associated infections in older adults, a population where immune function may be lower and where such infections are clinically challenging.

## 2. Materials and Methods

### 2.1. Participants and Sample Collection

Stool samples were collected from 20 healthy older individuals aged 60 to 75 years after they provided written informed consent. All participants were residents of the same city (Shijiazhuang, China). All participants completed a questionnaire reporting their basic information. Immunity-related details, such as autoimmune assessment scores, the incidence of cold and flu, average time needed to recover from cold and flu, and any allergies, were also recorded. The exclusion criteria included: (1) a history of acute infectious disease within the past four weeks; (2) any severe chronic disease requiring long-term medication (e.g., insulin-dependent diabetes, uncontrolled hypertension, heart failure, chronic kidney disease); (3) use of antibiotics, probiotics, or medications known to substantially alter gut microbiota (including proton pump inhibitors, metformin, NSAIDs, and laxatives) within the past three months; (4) gastrointestinal surgery within the past six months.

Based on the self-reported survey results, participants were categorized into an self- reported lower-immunity group (L, N = 10) and a normal-immunity group (H, N = 10) using the following criteria: individuals with a frequency of cold and flu ≥5 times per year and a recovery period >7 days were allocated to the L group; participants with cold/flu frequency ≤2 times per year were assigned to the H group to maximize contrast. No laboratory immune tests (e.g., lymphocyte counts, immunoglobulins, or inflammatory markers) were performed. The study protocol adhered to the ethics guidelines of the 1975 Declaration of Helsinki and was approved by the Ethics Committee of the 980 Hospital of the Joint Service Support Force of the People’s Liberation Army of China.

Participants received instructions regarding standard fecal sample collection procedures. Fresh fecal samples (~10 g) were collected using a sterile, dry stool collector and immediately stored at −20 °C. Samples were transported to the laboratory within 2 h of collection and stored thereafter at −80 °C until analysis.

### 2.2. Isolation and Enumeration of E. coli

The three-tube, five-dilution most probable number (MPN) method was employed to enumerate *E. coli* based on the protocol reported by [18], with some modifications. Briefly, triplicate aliquots of fecal samples (0.1–0.000001 g) were resuspended in tubes containing 10 mL Lauryl Sulfate Tryptose (LST, Beijing Land Bridge Technology Co., Ltd., Beijing, China) broth and incubated at 37 °C for 48 h. The MPN was calculated based on the number of tubes showing positive reactions (bubbles). One loop of positive LST tubes was transferred into 10 mL *E. coli* broth (EC, Beijing Land Bridge Technology Co., Ltd., China) and incubated at 44.5 °C for 48 h. The cultures were then streaked onto Eosin Methylene Blue (EMB, Beijing Land Bridge Technology Co., Ltd., China) plates and incubated at 37 °C for 18–24 h. Up to five presumptive *E. coli* colonies were selected from the EMB plates and verified using the indole, methyl red, Voges–Proskauer, and sodium citrate test (IMViC; Beijing Land Bridge Technology Co., Ltd., Beijing, China). The MPN per gram value of each sample was acquired by using the FDA-BAM MPN table [19]. For this assay, *E. coli* ATCC25922 and sterile buffered peptone water was used as the positive and negative control, respectively.

All picked colonies were subjected to phenotypic characterization, including colony morphology on EMB, IMViC tests, antimicrobial susceptibility, and biofilm formation assay. Colonies originating from the same sample that displayed identical profiles across all four phenotypic criteria were considered redundant and were discarded. Only phenotypically distinct isolates per sample were retained for further analysis. After this redundancy filtering, a total of 70 *E. coli* isolates were obtained from all samples combined and used for downstream reporting.

### 2.3. Antimicrobial Susceptibility and Biofilm Formation Capacity of E. coli

The Kirby–Bauer disc diffusion method was employed to assess the susceptibility of 70 *E. coli* isolates to 14 commonly used antibiotics (Table 1) [20]. Briefly, a 0.5 McFarland suspension of each isolate was streaked across the surface of a Mueller–Hinton agar (MHA, Beijing Land Bridge Technology Co., Ltd., China) plate. Antibiotic discs (Thermo Fisher Scientific Oxoid, Waltham, MA, USA) were aseptically placed onto the MHA plates, with a distance of at least 3 cm between their centers. After 24 h of incubation at 37 °C, the zones of inhibition around the discs were measured. For this assay, *E. coli* ATCC25922 was used as the quality control strain. The susceptibility and resistance profiles of each isolate were interpreted according to the Clinical and Laboratory Standards Institute (CLSI) M100, 33rd edition [20]. Isolates categorized as intermediate were considered susceptible in the present study and were not included in multidrug resistance and prevalence of resistance.

Biofilm formation by *E. coli* isolates was quantitatively assessed using the crystal violet staining method [21], with some modifications. A 100 μL aliquot of an overnight *E. coli* culture (adjusted to 0.5 McFarland) was added to the wells of a 48-well microplate containing 900 μL LB broth. The control wells were not inoculated with *E. coli*. The plates were incubated at 37 °C for 72 h. After incubation, the plates were rinsed with sterile phosphate-buffered saline (PBS) twice to remove unattached loose cells, fixed with methanol, dried at 60 °C for 30 min, and stained with 1% crystal violet for 30 min. The plates were then rinsed again with PBS, air-dried, and treated with 33% acetic acid. The optical density (OD) of each well was measured at 570 nm using a microplate reader (BIO-DL, Shanghai, China). The assay was repeated through three independent biological experiments, each performed in triplicate. For each biological replicate, the mean optical density (OD) of the three technical wells was calculated. The three biological replicate means were then used to determine the final OD ± standard deviation (SD) and for all statistical comparisons. The biofilm-forming capacity was categorized based on the cutoff optical density (ODc), as follows: none (OD ≤ ODc), weak (ODc < ODs ≤ 2 × ODc), moderate (2 × ODc < ODs ≤ 4 × ODc), and strong (4 × ODc < ODs) [22]. An MDR strain with strong biofilm formation capacity (L5-1) was selected for a proof-of-concept study in subsequent experiments.

### 2.4. Effect of Lactobacillus on E. coli Biofilms

#### 2.4.1. Bacterial Cultures

Five *Lactobacillus* strains—*L. plantarum* B22 (B22), *L. paracasei* 02 (LP02) (isolated and stored in our lab), *L. plantarum* 45 (LP45), *L. paracasei* L475 (L475), and *L. rhamnosus* 863 (LR863) (obtained from Hebei iNatural Biotech Co., Ltd., Shijiazhuang, China)—were used in this study. Preliminary experiments showed that these strains were capable of tolerating acidic pH (2.5) and bile salts up to concentrations of 0.5% (Supplementary Figure S1). Additionally, they showed great antibacterial activity against foodborne pathogens, such as *Salmonella*, indicating their significant probiotic potential. All *Lactobacillus* strains and *E. coli* L5-1 were stored at −80 °C and then activated in MRS broth and LB broth (Beijing Land Bridge Tech Co., Ltd., China), respectively, at 37 °C for 24 h. For preparation of *Lactobacillus* cell-free supernatants (CFSs), each activated *Lactobacillus* culture was adjusted to 0.5 McFarland turbidity, and 1 mL of this standardized suspension was inoculated into 100 mL fresh MRS broth (1%, *v*/*v*) and incubated at 37 °C for 24 h. The CFSs of *Lactobacillus* were prepared by centrifuging the cultures at 10,000× *g* and 4 °C for 5 min and filtering through a 0.45-μm syringe filter (Advantec, Tokyo, Japan). These CFS samples were then stored at −20 °C until further use.

#### 2.4.2. Biofilm Inhibition and Eradication Assays

The inhibitory capacity and eradication effect of *Lactobacillus* CFSs against *E. coli* biofilms were assessed using the crystal violet assay, with some modifications [23]. To determine the inhibitory effect, 20 µL of the *E. coli* suspension and 20 µL of the *Lactobacillus* CFS were added to a sterile 96-well polystyrene microplate along with 180 µL LB broth. Plates were incubated at 37 °C for 12, 24, and 48 h. Meanwhile, the control group was incubated with 20 µL PBS instead of *Lactobacillus* CFS.

For the eradication assays, 20 µL suspensions of overnight *E. coli* cultures were incubated in 180 µL LB broth for 48–72 h to allow biofilm formation. After washing with PBS, 200 µL of each *Lactobacillus* CFS was added, and the tubes were incubated at 37 °C for 1, 2, and 4 h. The control group was incubated with 200 µL PBS.

For both the inhibition and eradication assays, biofilm formation was determined as previously described (Section 2.3). All experiments were performed with three independent biological replicates. For each biological replicate, three technical replicates (i.e., three parallel wells per condition within the same microplate) were measured. The inhibition and eradication rates were calculated using the following formula:

(1) $$\mathrm{Inhibition}\;\mathrm{Rate}\;\mathrm{or}\;\mathrm{Eradication}\;\mathrm{Rate}\;(\%)=\frac{\left(OD\text{\_}control-OD\text{\_}experiment\right)}{OD\text{\_}control}$$

To evaluate the effect of *Lactobacillus* CFS on the viable counts of *E. coli* biofilms, the initial concentration of *E. coli* was adjusted to 8 log CFU/mL. *E. coli* strains were cultured in TSB alone (as a control) or in TSB supplemented with different *Lactobacillus* supernatants (100 µL CFS + 80 µL TSB + 20 µL bacterial suspension) and incubated at 37 °C for 48 h. Bacterial growth was determined by direct spread plating onto EMB agar plates. The same method was used for *E. coli* biofilm eradication, except that *Lactobacillus* CFS was added to pre-formed *E. coli* biofilms instead of being co-incubated with the bacterial culture. All samples were tested in duplicate, and each experiment was performed twice.

### 2.5. Determination of the Minimal Biofilm Inhibitory Concentration (MBIC) and Minimum Biofilm Eradication Concentration (MBEC)

*Lacticaseibacillus paracasei* L475 was selected for further investigation due to its higher inhibition and eradication rates. The MBIC was determined as described by Zheng et al. [24], with slight modifications. Briefly, 100 µL of *E. coli* suspensions (8 log CFU/mL) were added to each well of a 96-well plate along with 100 µL of serial two-fold dilutions of *Lactobacillus* CFS (1.6% to 100%). The plates were then incubated at 37 °C for 24 h. The MBIC was defined as the lowest concentration at which no visible turbidity was detected.

To determine the MBEC, the crystal violet staining method was employed, as reported by Zhang et al. [25]. First, *E. coli* biofilms were cultured in 96-well plates at 37 °C for 48 h. After removing the culture medium, 200 µL of serial two-fold dilutions of *Lactobacillus* CFS were added to each well and incubated at 37 °C for 8 h. The biofilm biomass was determined using crystal violet staining, as previously described (Section 2.3). The minimum concentration that yielded no significant change in OD values when compared with the control (wells without *E. coli* biofilms) was considered the MBEC.

### 2.6. Effect of CFS on EPS Production in E. coli Biofilm

The EPS production in *E. coli* biofilms was measured during the inhibition and eradication phases following *L. paracasei* L475 CFS treatment using colorimetric methods [23]. Briefly, during the inhibition assay, *E. coli* and *Lactobacillus* CFS (1/2 MBIC, 2 MBIC) were co-incubated in 48-well microplates at a ratio of 1:1. After incubation at 37 °C for 6 h, 12 h, and 24 h, the culture medium was carefully removed. Each well was gently washed, and 1.2 mL of PBS was added. The samples were sonicated for 5 min and centrifuged at 10,000× *g* for 10 min. The supernatant was filtered through a 0.45 μm filter. A similar method was used during *E. coli* biofilm eradication, except that *Lactobacillus* CFS was added to pre-formed *E. coli* biofilms instead of the co-incubation step.

The concentrations (μg/mL) of extracellular polysaccharides and protein were measured using the phenol–sulfuric acid method [26] and Coomassie brilliant blue method [25], respectively. To quantitatively examine the extracellular polysaccharides and protein, 50 μg/mL fluorescein isothiocyanate-labeled Concanavalin A (FITC-ConA, Xi’an Qiyue Biotechnology Co., Ltd., Xi’an, China) and 1 μM Rhodamine B (Beijing Solarbio Science & Technology Co., Ltd., Beijing, China) were added to the samples (as prepared above), respectively, and incubated in the dark for 10 min. After the removal of the staining solution and gentle washing, the samples were rinsed with sterile water, dried, and examined using a fluorescence microscope (Axio Imager A2, Carl Zeiss, Jena, Germany). Three randomly selected fields of view were acquired per sample. One representative field of view from the three was selected for image presentation. All image acquisition settings were kept constant across all treatment groups. Quantification of fluorescence intensity was performed using ImageJ version 1.45 software by an investigator who was blinded to the treatment allocation.

### 2.7. Scanning Electron Microscopy (SEM)

The inhibition and eradication effects of *Lactobacillus* CFS (1/2 MBIC/MBEC and 2 MBIC/MBEC) against *E. coli* biofilms were tested in a 6-well microplate with glass coverslips using the method described in Section 2.4.2. After incubation, the biofilms on the glass coverslips were fixed with 2.5% (*v*/*v*) glutaraldehyde for 1 h, followed by three rinses with PBS. Gradient dehydration was performed using ethanol concentrations (*v*/*v*) of 50%, 70%, 80%, 90%, and 100% for 10 min each, followed by immersion in isoamyl acetate for 20 min. After freeze-drying and gold sputter coating, the samples were observed using a scanning electron microscope (Hitachi, Tokyo, Japan, SU8600). SEM images shown are representative of three independent biological replicates with similar results. For each replicate, at least two randomly selected fields of view per sample were examined at 2000× and 20,000× magnification.

### 2.8. Confocal Laser Scanning Microscopy (CLSM)

The inhibition and eradication effects of *Lactobacillus* CFS (1/2 MBIC/MBEC and 2 MBIC/MBEC) against *E. coli* biofilm were tested in a confocal dish using the method described in Section 2.4.2. The confocal dishes were washed twice with PBS and dried for 20 min. To distinguish between live and dead cells, SYTO-9 and propidium iodide (PI) dyes were added at concentrations of 2 μM and 10 μM, respectively. The biofilms were then incubated in the dark at 25 °C for 10 min. After removing the staining solutions, the samples were examined using CLSM (Leica, Wetzlar, Germany, TCS Sp8). To quantify biofilm thickness, Z-stack images were acquired for each sample. Thickness was estimated as the distance from the first Z-plane with attached cells to the last plane with detectable fluorescence and were processed using BiofilmQ version 1.0.1 software.

### 2.9. Data Analysis

All experiments were performed in triplicate. Data were analyzed using IBM SPSS software v26.0 (IBM Inc., Armonk, NY, USA). For comparisons of phenotypic traits (e.g., antimicrobial resistance prevalence) between the H and L groups, chi-square tests were performed at the isolate level (33 isolates in group H, 37 isolates in group L). Because multiple isolates from the same participant may not be statistically independent, these isolate-level analyses are considered exploratory and descriptive. Meanwhile, quantitative data were analyzed using one-way analysis of variance (ANOVA). Post hoc pairwise comparisons were performed using Tukey’s HSD test, which controls the family-wise error rate for those specific comparisons. Statistical significance was set at *p* < 0.05.

## 3. Results and Discussion

### 3.1. Demographic Characteristics of Study Participants

In total, 20 older adults were included in the study (Table 2). These subjects were classified into a self-reported lower-immunity group (L, N = 10) and a normal-immunity group (H, N = 10) based on their self-assessment questionnaire, the incidence of cold and flu, and the average time to recovery. Specifically, individuals with a frequency of cold and flu ≥5 times a year and a recovery period >7 days were allocated to the self-reported lower-immunity group. Participants with cold/flu frequency ≤2 times per year were assigned to the comparison (H) group to maximize contrast. No laboratory immune tests were performed. There was no significant difference in gender distribution (χ^2^ = 0.0, *p* = 1.0), age (t = 1.70, *p* = 0.467), and body mass index (t = −0.62, *p* = 0.404) between the L and H groups. However, the incidence of cold and flu per year and the time to recovery were significantly lower in the H group than in the L group (*p* < 0.05). Meanwhile, immunity-related self-assessment scores were significantly higher in the H group than in the L group.

### 3.2. MPN-Based Enumeration of E. coli

**Figure 1:**
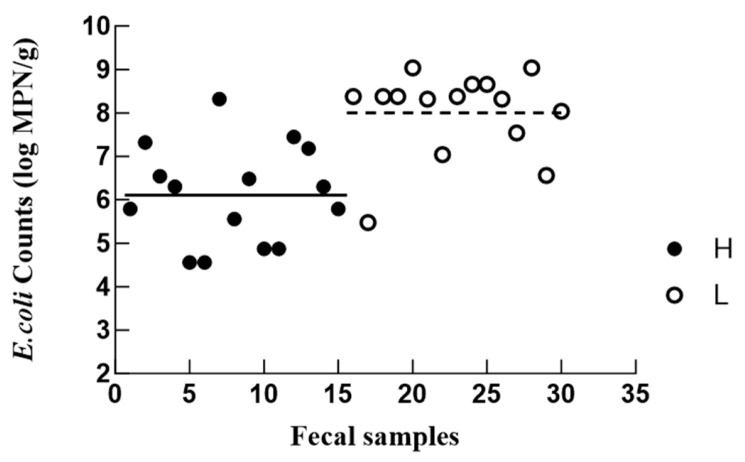
Most probable number (MPN)-based enumeration of *E. coli* from older individuals with different immunity levels. H: Normal-immunity group; L: self-reported lower-immunity group. The line represents the average MPN counts of *E. coli* in each group.

Among all the isolates, only phenotypically distinct isolates per sample were retained. After this filtering, a total of 70 unique *E. coli* isolates were selected and used for reporting, including 33 strains from the H group and 37 strains from the L group. The counts of fecal *E. coli* varied widely across the participants, ranging from 4.56 log MPN/g to 9.04 log MPN/g (Figure 1), in line with the previously reported range of 2–9 log CFU/g [27]. Despite inter-individual variability, the mean *E. coli* counts were significantly higher in the L group (7.89 ± 0.25 log MPN/g) than in the H group (6.04 ± 0.37 log MPN/g) (*p* < 0.05). This difference raises the possibility that fecal *E. coli* abundance may be associated with immune-related health status in older adults, but further studies with larger cohorts and stricter confounder control are needed. Although recent evidence has revealed that intestinal *E. coli* colonization is greater in older individuals [3,28,29], little attention has been paid to the gut microbiota variations in older individuals with different levels of immunity. Previous reports of a higher relative abundance of *E. coli* and Enterobacteriaceae in frail [30] and hospitalized [31] older patients provide a relevant precedent, particularly given the established role of immune dysfunction in frailty [32]. As a prominent member of the Enterobacteriaceae family, *E. coli* has been implicated in various geriatric disorders, including inflammatory bowel disease, urinary tract infections, and Crohn’s disease [33,34,35]. This association supports our hypothesized link between *E. coli* abundance and the health and immune status of older adults. The observed inter-individual variability within the H and L groups could be attributed to confounding factors such as diet and exercise [36].

Although rigorous inclusion and exclusion criteria were applied, potential confounding factors (e.g., diet, hygiene, living environment) could not be fully excluded and may have influenced our results—a common challenge in gut microbiota studies. Furthermore, while phenotypically redundant isolates were removed per sample, multiple isolates from the same participant may remain correlated. Therefore, reported *p*-values likely underestimate variability. Future studies with larger cohorts should use participant-level analysis or clustered methods (e.g., mixed-effects models). Despite these limitations, this initial analysis provides preliminary observations that may inform future investigations of gut microbial markers of immune dysfunction in older adults.

### 3.3. Antimicrobial Susceptibility Profile of E. coli Isolates

**Figure 2:**
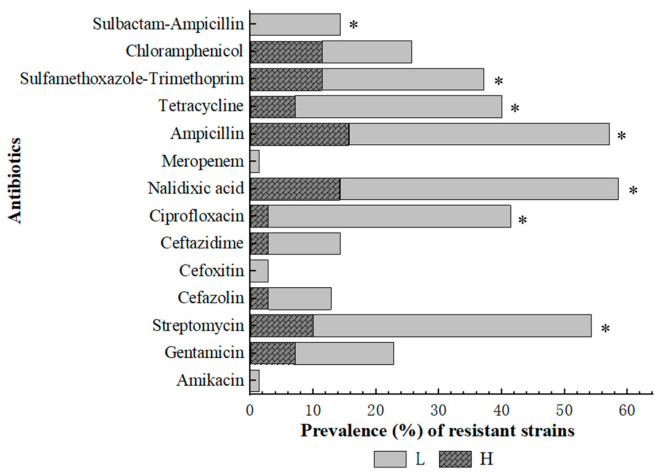
Prevalence of strains resistant to different antibiotics (out of the total number of *E. coli* strains isolated from older individuals). * indicates that *E. coli* isolates from the self-reported lower-immunity group (L, N = 37) were significantly (*p* < 0.05) more likely to be resistant to certain antibiotics than those from the normal-immunity group (H, N = 33).

The development of antibiotic resistance in *E. coli*, which is a clinically significant pathogen, is a serious public health challenge. Among the 70 isolates obtained in this study, 56 (80.0%) were resistant to at least one of the tested antimicrobial agents. Herein, resistance was most commonly detected against Nalidixic acid (58.6%), followed by Ampicillin (57.1%), Streptomycin (54.3%), Ciprofloxacin (41.4%), and Tetracycline (40.0%). Additionally, 37.1% of the isolates were resistant to Sulfamethoxazole-Trimethoprim, 25.7% to C, 22.9% to Gentamicin, 14.3% to Ceftazidime and Sulbactam-Ampicillin, 12.9% to Cefazolin, and 2.9% to Cefoxitin. One isolate (1.4%) was resistant to both Meropenem and Amikacin (Figure 2). Furthermore, 20 isolates (60.6%, N = 33) from the H group and 36 (97.3%, N = 37) from the L group were resistant to at least one antibiotic (*p* < 0.05, Figure 2). In addition, *E. coli* isolates from the L group were significantly (*p* < 0.05, Figure 2) more likely to be resistant to Streptomycin (83.8% vs. 21.2%), Ciprofloxacin (73.0% vs. 6.1%), Nalidixic acid (83.8% vs. 30.3), Ampicillin (78.4% vs. 33.3%), Tetracycline (62.2% vs. 15.2%), Sulfamethoxazole-Trimethoprim (48.6% vs. 24.2%), and Sulbactam-Ampicillin (27.0% vs. 0) than those from the H group.

*E. coli* isolates resistant to at least one agent across three or more antibiotic classes were considered to show MDR. At the participant level, five in the H group carried at least one MDR isolate, while all the participants in the L group carried MDR isolates (Table S1). At the isolate-level, 41/70 isolates (58.6%) were MDR, with resistance to 3–8 of the tested antibiotics (Table 3). Seven strains each were resistant to three and four classes of antibiotics. The most frequent (27/41) multidrug resistance pattern involved resistance to five or six classes of antibiotics simultaneously. Of the isolated strains, only one was resistant to seven classes of antibiotics. The MDR isolates were all resistant to a combination of aminoglycosides (streptomycin or gentamycin), quinolone (nalidixic acid or ciprofloxacin), cephalosporins (cefazolin, cefoxitin, or ceftazidime), tetracyclines (tetracycline), and sulfonamides (sulfamethoxazole-trimethoprim). The prevalence of MDR isolates was significantly higher (*p* < 0.05) in the L group (91.7%, 33/36) than in the H group (24.2%, 8/33).

Increasing antibiotic resistance in *E. coli* has emerged as a global health issue. Although the exact reported patterns of antibiotic resistance in *E. coli* have varied depending on the study population (e.g., age, gender), region, and pathological scenario, resistance to penicillins and quinolones has remained consistently high [5,37,38]. Similarly, a high rate of resistance to penicillins (i.e., AMP) and quinolones (i.e., NA and CIP) was detected in the present study. However, there were some differences in the results of the present study and previous reports. Notably, the percentage of *E. coli* isolates resistant to tetracycline (40%) was much lower than that reported in previous studies (60–92.9%) [9,39]. The pattern and extent of antibiotic resistance in *E. coli* are influenced by factors such as the presence of multiple strains in a single sample, sources of isolation, geographical location, and recent antibiotic use. Notably, in this study, *E. coli* strains were more likely to be resistant to the third-generation antibiotics (CAZ) than to earlier agents (e.g., FOX). This dramatic increase in resistance to third-generation cephalosporins has been reported previously in *E. coli* [40] and has been attributed to the extensive use of these broad-spectrum, high-efficacy antimicrobials [41]. These resistance patterns may potentially be associated with an increased risk of mortality, contributing to a serious global health burden [42].

In recent years, several studies have confirmed the increase in MDR across *E. coli* isolates from humans, animals, and food products. In the current study, 65.8% of the MDR isolates were simultaneously resistant to five or six classes of antibiotics, with the most frequent MDR pattern involving resistance to penicillin–aminoglycosides–quinolones–tetracyclines. Similarly, previous studies have reported that *E. coli* isolates from animal and clinical samples are often resistant to five antibiotic classes, including penicillins, aminoglycosides, tetracycline, and quinolones [43,44,45]. Our results highlight the serious issue of MDR *E. coli* carriage in fecal samples from volunteer older adults, which may cause *E. coli* to evolve into a super bacterium and pose a serious risk to public health [46].

In the present study, both the antibiotic resistance rate and MDR frequency of *E. coli* were significantly higher in the L group than in the H group. This indicated a correlation between the immune status of older adults and antimicrobial resistance in their gut microbiota. These results corroborate previous findings regarding a higher MDR rate in *E. coli* isolates from hospitalized and low-immunity patients when compared to those from healthy controls [47,48,49]. Several speculative explanations for this association warrant consideration in future research. First, although not directly measured in our cohort, individuals with poor immunity are more likely to be hospitalized or undergo repeat hospitalization, and therefore, they may be exposed to antimicrobials more frequently. Such selective pressure could lead to a higher prevalence of resistant strains in the gut in these individuals [50]. In addition, risk factors for MDR in older adults, such as immunosenescence, polypharmacy, frailty, and frequent hospitalizations [51,52], also likely underpin this relationship. However, because our study did not collect data on hospitalization history, antimicrobial use, or frailty status, these interpretations remain speculative.

### 3.4. Biofilm Formation Capacity of E. coli Isolates

**Figure 3:**
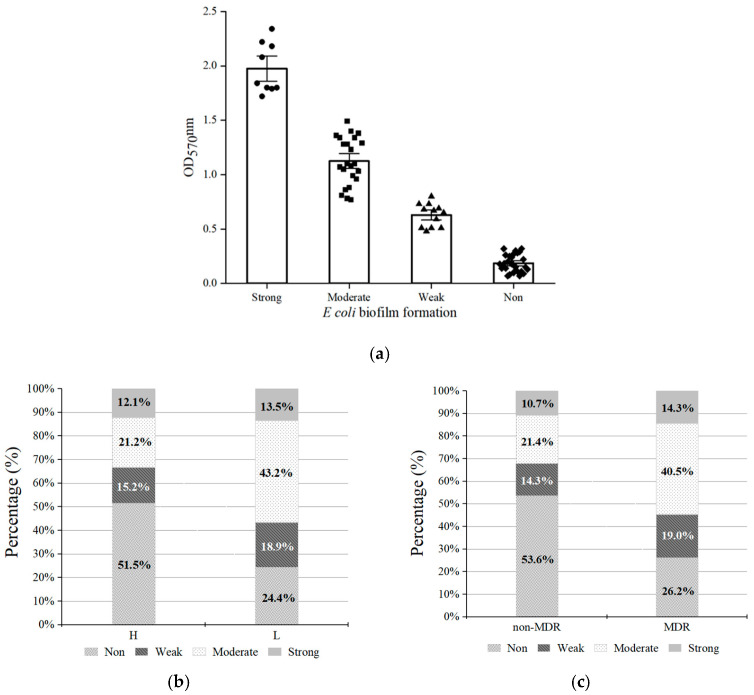
Biofilm formation capacity of *E. coli* strains recovered from older individuals. (**a**) Overall biofilm formation capacity; (**b**) biofilm phenotypes among isolates from individuals with normal immunity (H) and self-reported low immunity (L); (**c**) biofilm phenotypes among isolates with multidrug resistance (MDR) and without MDR.

Biofilm formation by *E. coli* isolates was assessed using the crystal violet assay. Of the 70 isolates, 44 (62.9%) exhibited the ability to produce biofilms. These isolates were classified as strong (12.7%, 9 isolates), moderate (32.8%, 23 isolates), and weak (17.1%, 12 isolates) biofilm producers (Figure 3a). Among the 37 isolates from the L group, 28 (75.7%) were biofilm producers. This proportion was significantly higher than that observed in the H group (48.5%, χ^2^ = 5.52, *p* < 0.05, Figure 3b), indicating a potential correlation between biofilm formation and host immune status. In line with this, 30 of the 41 MDR *E. coli* isolates (73.2%) were found to be biofilm producers, and this rate was significantly higher than that among non-MDR *E. coli* isolates (46.4%, χ^2^ = 5.38, *p* < 0.05, Figure 3c).

Biofilms are associated with the long-term persistence of bacteria across diverse environments. In this study, 62.9% of *E. coli* isolates recovered from older individuals were found to be biofilm producers, with moderate biofilm production being the most common phenotype. This was consistent with previous reports indicating that although most *E. coli* isolates can form biofilms, the majority produce weak or moderate biofilms [53,54]. However, some studies have reported a higher prevalence of strong biofilm-producing strains [55,56]. These discrepancies could be attributed to methodological differences between studies, geographical variation, and the sources of isolated strains [57]. Furthermore, in this study, isolates from self-reported low-immunity older individuals exhibited significantly greater biofilm-forming capacity than those from healthy older individuals. This finding was consistent with previous reports regarding more extensive [58] and altered [59] biofilm formation in patients with chronic rhinosinusitis and gastrointestinal diseases, respectively, when compared to healthy controls. Such enhanced biofilm formation capacity in isolates from self-reported low-immunity individuals may represent a key survival strategy that allows bacteria to adapt to the host’s pathological environment. One possible explanation is that the chronic inflammatory state in such hosts [60], wherein inflammatory factors activate signaling molecules such as cyclic di-GMP, may promote biofilm formation in gut microbiota [61].

The relationship between biofilm formation and antibiotic resistance has remained a major focus of research. According to the current literature, the prevalence of biofilm-associated MDR ranges from 17.9% to 100% [13]. In the present study, MDR *E. coli* strains were significantly more likely to be biofilm producers. Similarly, in a previous study from Uganda, biofilm-forming clinical *E. coli* isolates were more frequently found to exhibit MDR phenotypes (64%) than nonbiofilm producers (36%) [62]. In addition, strong associations between MDR and biofilm production have also been observed in various microorganisms isolated from clinical samples [55], medical devices [63], and the food supply chain [64]. Biofilm formation is a major mechanism of drug resistance, contributing to MDR by limiting antibiotic penetration, enabling quorum sensing, increasing cellular density and persistent cell populations, and promoting the horizontal transfer of resistance genes within the biofilm matrix [65,66]. These observations indicate that biofilm formation in the tested *E. coli* isolates could potentially be linked to MDR, complicating eradication. This highlights the need to develop intervention strategies against biofilm-associated MDR strains. Therefore, based on its MDR profile and strong biofilm-forming capacity, *E. coli* isolate L5-1 was selected for a proof-of-concept study in subsequent experiments.

### 3.5. Effect of Lactobacillus on E. coli L5-1 Biofilms

**Figure 4:**
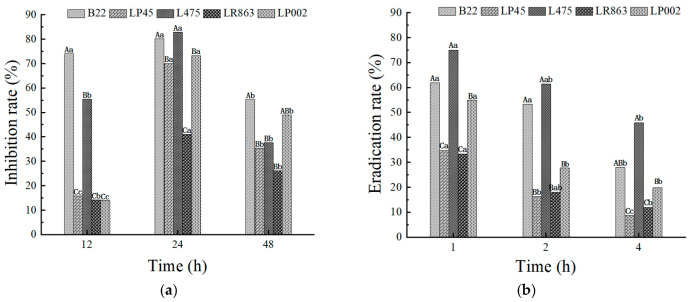
Antibiofilm activity of the tested *Lactobacillus* strains against *E. coli* L5-1. (**a**) Inhibition assay; (**b**) eradication assay. Different uppercase letters on the columns denote significant differences (*p* < 0.05) in the inhibition/eradication rate among different *Lactobacillus* strains at the same time point; different lowercase letters on the columns denote significant differences in the inhibition/eradication rate of the same *Lactobacillus* strain at different treatment time points.

The ability of the CFS from *Lactobacillus* strains to both inhibit the formation and eradicate pre-formed *E. coli* L5-1 biofilms was quantitatively assessed using crystal violet assays. The results demonstrated a *Lactobacillus* strain-specific variability in efficacy, with biofilm inhibition rates ranging from 14.2% to 82.9% and eradication rates ranging from 8.6% to 75.0% (Figure 4). Such variations have been noted in several previous studies [15,67]. In the present study, L475 (82.9%) and B22 (80.2%) provided the highest inhibition rates at 24 h (*p* < 0.05), followed by LP45 (70.1%) and LP002 (73.3%). In contrast, LR863 (41.0%) produced the lowest inhibition rate (Figure 4a). Similarly, L475 (75.0%) and B22 (61.9%) induced the highest eradication rate after 1 h of treatment, followed by LP002 (55.1%), while LP45 (34.8%) and LR863 (33.4%) produced the lowest eradication rate (Figure 4b). This broad spectrum of activity was consistent with the literature. Previous studies have shown that the antibiofilm potential of lactobacilli is a strain-specific trait that is intrinsically linked to the unique metabolic activity and cellular components of each strain [68,69]. Thus, the variation detected in this study could be due to the different concentrations and release kinetics of bioactive antibiofilm metabolites in different *Lactobacillus* strains [70,71].

Regarding the effect of CFSs on viable counts of *E. coli*, all tested CFSs reduced the growth of *E. coli* biofilms below the detection limit (0.7 log CFU/mL) within 32 h (Figure S2a). Among them, L475 and LP45 exhibited the strongest inhibitory effect, achieving complete inhibition of *E. coli* biofilm formation at 16 h. For the eradication assay, all tested CFSs reduced *E. coli* biofilms below the detection limit (0.7 log CFU/mL) within 24 h, showing faster efficacy than the inhibition assay, which is consistent with the crystal violet assay. B22, LP45, and L475 performed most effectively, achieving complete eradication of *E. coli* biofilms at 12 h, followed by LR863, which completely eradicated biofilms at 16 h (Figure S2b). The superior performance of L475 across both assays highlighted the value of this strain as a particularly promising candidate for controlling biofilms. Both the MBIC and MBEC of L475 CFS for *E. coli* L5-1 biofilms were calculated to be 12.5%.

The antibiofilm activity of *Lactobacillus* CFSs is typically attributed to a combination of primary metabolites such as organic acids (lactic, acetic) and hydrogen peroxide, which create a low-pH environment, as well as more specialized compounds such as bacteriocins and biosurfactants [72,73]. The exceptional potency of L475 observed in this study raises the hypothesis that its CFS may contain a particularly effective combination of such agents, although we cannot rule out that the effect is primarily due to organic acids and low pH because the CFS was not neutralized. In line with this hypothesis, Dasgupta et al. [74] reported that a lipopeptide biosurfactant produced by probiotic bacteria contributes to their antibiofilm capacity. Furthermore, CFSs may contain signaling molecules or enzymes that downregulate genes involved in biofilm development, including those involved in adhesion, quorum sensing, and matrix production [75]. The relatively poor performance of LR863 serves as a control, indicating that the observed antibiofilm activity is strain-specific rather than a generic property of all lactobacilli. However, without neutralized CFS, metabolomic or proteomic characterization of the CFS, the specific active compounds responsible for the potency of L475 remain unidentified.

In this study, with regard to biofilm inhibition, the strongest effect was observed after 24 h of co-incubation (Figure 4a). This suggested that the active compounds in the CFS do not merely kill planktonic bacterial cells but also actively interfere with the developmental pathways of the biofilm over an extended period. These effects could involve the downregulation of genes critical for adhesion, matrix production, and quorum sensing [75]. The lower rate of inhibition at 12 h likely reflected the initial “establishment” phase, where the biofilm mass was still low and the cumulative effect of the CFS was not fully obvious. By 48 h, the presence of persistent cells within the mature biofilm structure likely reduced the overall susceptibility, despite CFS treatment [76]. In contrast, the biofilm eradication assay revealed a rapid-action mechanism, with the most significant reduction in pre-formed biofilms obtained after 1 h of treatment with the *Lactobacillus* CFS (*p* < 0.05 vs. 2 h and 4 h, Figure 4b). This strongly implied that the primary mode of action against mature biofilms involves a direct and immediate physicochemical attack on biofilm structure. This is consistent with the action of biosurfactants and high-concentration organic acids, which quickly solubilize the EPS matrix and compromise cell membrane integrity [77]. In contrast, Carvalho, Mergulhão and Gomes [72] observed progressive biofilm eradication after CFS treatment. These inconsistencies could be due to the different amounts and concentrations of CFS used in their study.

A key limitation of the antibiofilm experiments is that only one MDR *E. coli* isolate (L5-1) was tested. The prevalence of MDR *E. coli* in the study population was high, but the genetic and phenotypic diversity among isolates is substantial. Therefore, whether the observed antibiofilm activity of L475 CFS extends to other MDR *E. coli* isolates from older adults remains unknown. Future studies should test a larger, well-characterized collection of clinical MDR *E. coli* isolates.

### 3.6. Effect of L475 CFS on EPS Production in E. coli L5-1 Biofilms

The EPS matrix is a critical structural and functional component of bacterial biofilms. Primarily consisting of polysaccharides, proteins, and extracellular DNA, the EPS matrix provides a stable architecture to biofilms, mediates surface adhesion, facilitates intercellular interactions, and acts as a protective barrier against environmental stressors such as antibiotics and sanitizers [25,78]. Consequently, inhibiting or eradicating EPS secretion is a key strategy for controlling biofilm production. In this study, the effects of L475 CFS on the key components of this matrix, i.e., polysaccharides and proteins, were investigated during both biofilm formation and the eradication of pre-formed *E. coli* L5-1 biofilms.

**Figure 5:**
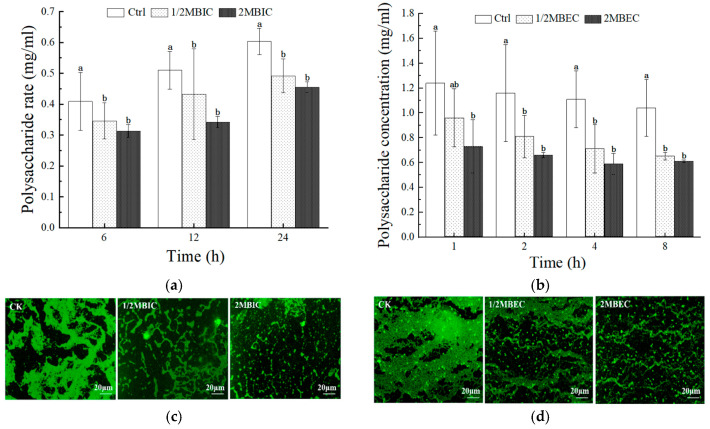
Inhibitory capacity (**a**) and eradication effect (**b**) of the *Lacticaseibacillus paracasei* L475 cell-free supernatant on the formation of extracellular polysaccharides in *E. coli* L5-1 biofilms, accompanied by fluorescence microscopy images of extracellular polysaccharides during the inhibition (**c**) and eradication (**d**) assays. Different lowercase letters in the figure indicate significant differences among the treatments within the same time points (*p* < 0.05).

**Figure 6:**
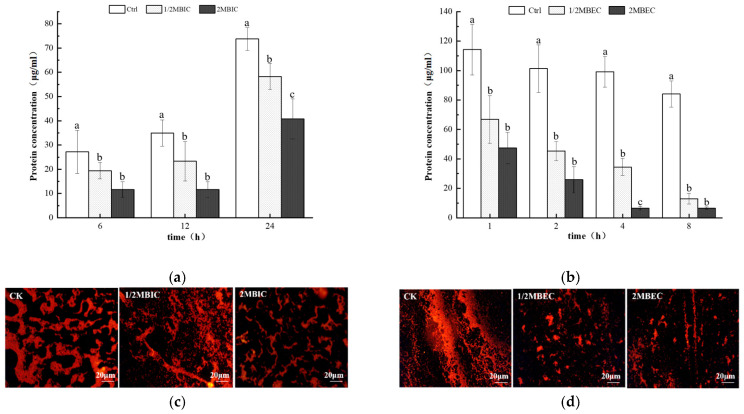
Inhibitory capacity (**a**) and eradication effect (**b**) of the *Lacticaseibacillus paracasei* L475 cell-free supernatant on the formation of extracellular proteins in *E. coli* biofilms, accompanied by fluorescence microscopy images of extracellular proteins during the inhibition (**c**) and eradication (**d**) assays. Different lowercase letters in the figure indicate significant differences among the treatments within the same time points (*p* < 0.05).

A significant reduction in extracellular polysaccharide production was detected in developing *E. coli* L5-1 biofilms (at 6, 12, and 24 h) upon treatment with sub-inhibitory (1/2 MBIC) and inhibitory (2 MBIC) concentrations of L475 CFS, when compared to untreated controls (Figure 5a). However, no significant difference was detected between the effects of L475 CFS at 1/2 MBIC and 2 MBIC. A similar trend was observed in the eradication assay, wherein treating pre-formed *E. coli* L5-1 biofilms with L475 CFS (2 MBEC) for 8 h led to a 41.3% reduction in polysaccharide content (Figure 5b). Notably, a more pronounced effect was observed on the proteinaceous component of the EPS. After treatment with L475 CFS at 2MBIC for 24 h and at 2MBEC for 8 h, the secretion of extracellular proteins was reduced by 44.7% (Figure 6a) and 92.3% (Figure 6b), respectively. This disparity in reduction rates between polysaccharides (41.3%) and proteins (92.3%) in the eradication assay may raise the hypothesis that the CFS may contain proteolytic enzymes or specific metabolites that preferentially target proteinaceous adhesins and scaffold proteins more effectively than polysaccharides. However, future studies related to proteomic and metabolomic characterization of the supernatants would be helpful in identifying the actual active compound.

The quantitative findings were corroborated by fluorescence microscopy, which visually confirmed a reduction in biofilm area and weakening of fluorescence intensity after CFS treatment (Figure 5c,d and Figure 6c,d). This disruption of EPS production was consistent with accumulating evidence highlighting the role of this mechanism as a key contributor to the antibiofilm effects of lactobacilli. For instance, metabolites such as lipoteichoic acid and digestive enzymes, present in *Lactobacillus* CFS, have been found to disrupt EPS integrity [79,80]. Furthermore, a study by Park et al. [81] showed that metabolites from various *Lactobacillus* strains can effectively inhibit and eradicate biofilms produced by single and multi-species pathogens, mainly by compromising EPS production.

Interestingly, no significant difference in both polysaccharide and protein content was observed after the 1/2MBIC (MBEC) and 2MBIC (MBEC) treatments. Hence, within this dose range, CFS appeared to exert a dual effect by both killing bacteria and directly degrading EPS. The release of intracellular contents due to cell death was likely counteracted by EPS degradation, resulting in a net decrease in EPS and potentially creating a plateau of clearance efficacy. Furthermore, additional mechanisms, such as a reduction in the swimming capacity of bacteria, prevention of bacterial cell aggregation, and downregulation of genes related to biofilm formation, may have contributed to the inhibition and eradication of biofilms [16].

### 3.7. SEM and CLSM Analysis

**Figure 7:**
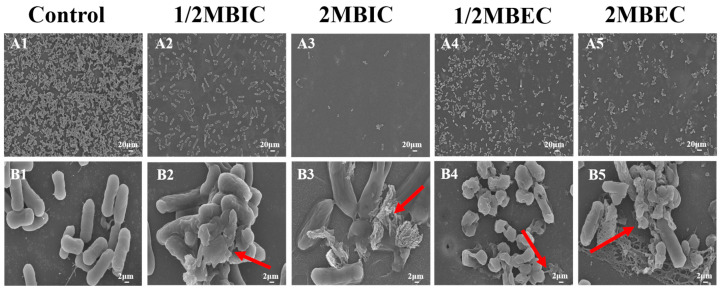
Scanning electron microscopy images of *E. coli* L5-1 biofilms after treatment with cell-free supernatant of *Lacticaseibacillus paracasei* L475. (**A1**,**B1**): Negative control; (**A2**,**B2**): 1/2 × MBIC and (**A3**,**B3**): 2 × MBIC by inhibition assay; (**A4**,**B4**): 1/2 × MBEC and (**A5**,**B5**): 2 × MBEC by eradication assay. Scale bars: first row = 20 μm; second row = 2 μm. Images are representative of three independent experiments.

The changes in the morphology of biofilms and *E. coli* L5-1 cells after treatment with L475 CFS on *E. coli* L5-1were evaluated using SEM and CLSM during the inhibition and eradication phases. SEM micrographs of untreated *E. coli* L5-1 biofilms (Figure 7(A1)) qualitatively revealed a well-established structure characterized by a dense network of cells embedded in a prominent reticular matrix with visible filamentous connections. In contrast, biofilms treated with L475 CFS exhibited a dispersed and fragmented structure. A marked reduction in cell aggregation was observed, along with a noticeable decrease in the density of surface-attached cells, especially following treatment with the 2MBIC and 2MBEC doses. Importantly, CFS treatment induced severe structural disruption, with extensive areas of cell lysis, membrane breakage, and the leakage of intracellular contents (arrows in Figure 7). This loss of cellular integrity suggested that the L475 CFS not only cleared the protective EPS matrix but also exerted a direct bactericidal effect on the resident cells within the biofilm. These observations were consistent with a previous report from Spaggiari et al. [77] who found that membrane disruption was a key contributor to the activity of *L. rhamnosus* CFS against *Candida albicans* biofilms, suggesting the presence of a broad-spectrum membrane-disrupting active component in the CFS.

**Figure 8:**
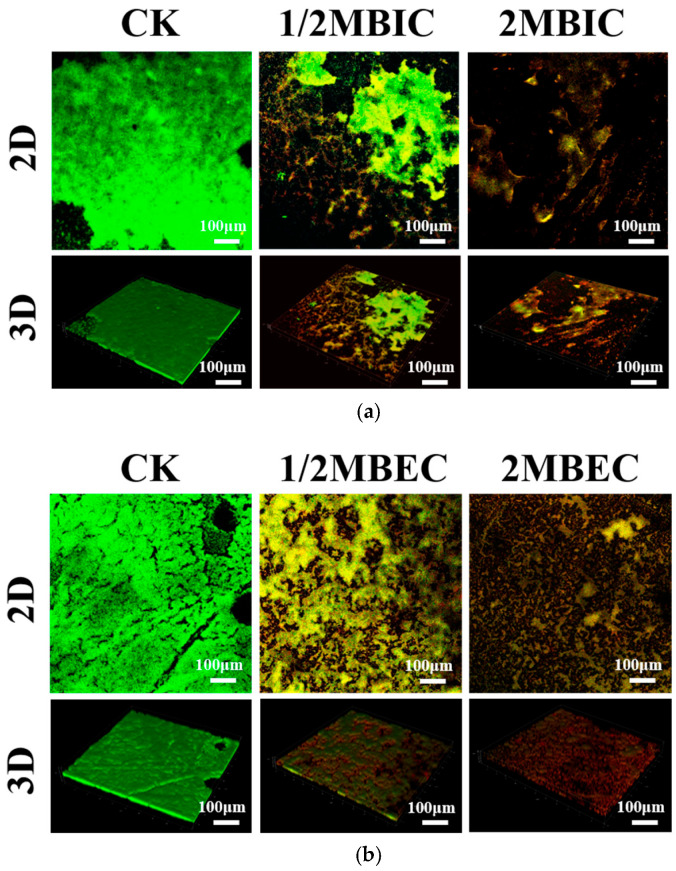
CLSM images of *E. coli* L5-1biofilms inhibited (**a**) and eradicated (**b**) by the cell-free supernatant of *Lacticaseibacillus paracasei* L475. Live bacteria are stained green, while dead bacteria are stained red. CK = Control. Images are representative of three independent experiments with similar results.

Subsequently, CLSM provided additional insights into the viability and structural features of the *E. coli* L5-1 biofilms (Figure 8). Live/dead staining (Syto 9/PI) in the control group revealed a predominance of green fluorescence, indicating that the biofilm was viable and healthy and mainly contained live cells. Following treatment with L475 CFS, a significant shift in fluorescence was observed, with the interiors fluorescing green and the exterior exhibiting red fluorescence. This dual-staining pattern was characteristic of cells with damaged or compromised membranes, confirming the membrane-disruptive effect observed using SEM. Visual inspection of CLSM images suggested a reduction in biofilm thickness after treatment, but because quantitative thickness measurements were not performed, this observation is descriptive only. This reduction was a direct consequence of both cell death and the dissolution of the EPS matrix. In line with these findings, Jun Meng [82] discovered that the antibacterial activity of *Lactobacillus* species can be attributed to the release of antimicrobial metabolites and inhibitory compounds that can target bacteria such as *E. coli*. Interestingly, *Lactobacillus* metabolites have also been shown to decrease the fatty acid content of bacterial cell membranes and alter membrane protein structures, causing the leakage of essential molecules and ions and ultimately inducing cell death. A previous study reported that the inhibitory effect of *L. plantarum* CFS is mediated by the release of bacteriocin [83]. The positively charged amino acid residues of bacteriocin can facilitate pore formation in target bacterial cells and exert electrostatic forces on cell membranes, causing the leakage of electrolytes and subsequently triggering cell lysis.

## 4. Conclusions

This study provides a systematic comparison of fecal *E. coli* carriage, antimicrobial resistance, and biofilm-forming capacity among community-dwelling older adults with different self-reported immune statuses, and evaluates the antibiofilm activity of five *Lactobacillus* cell-free supernatants against a representative multidrug-resistant and strongly biofilm-forming *E. coli* isolate (L5-1). Older adults with self-reported lower immune status (frequent cold/flu and prolonged recovery) had significantly higher fecal *E. coli* counts (7.89 vs. 6.04 log MPN/g), higher rates of resistance to at least one antimicrobial agent (97.3% vs. 60.6%), and a higher prevalence of multidrug resistance (91.7% vs. 24.2%) compared to those in the self-reported normal-immunity group. Among the 70 selected isolates, 62.9% were biofilm producers, and MDR isolates were significantly more likely to form biofilms than non-MDR isolates (73.2% vs. 46.4%). Mechanistically, L475 CFS reduced the extracellular polysaccharide and protein components of the EPS matrix, with a more pronounced reduction in proteins (92.3% at 2 × MBEC for 8 h) than in polysaccharides (41.3%). SEM and CLSM qualitatively confirmed that L475 CFS disrupted biofilm architecture, induced cell membrane damage, and caused cell lysis.

Several limitations of the present study should be noted. First, immune status was defined by self-report without laboratory markers, and the small sample size with multiple isolates per participant precludes definitive statistical inference. Second, all antibiofilm experiments were conducted using only one MDR *E. coli* isolate, and the CFS was not neutralized, leaving the relative contributions of acidity versus strain-specific metabolites undetermined. Third, EPS measurements were not normalized to biofilm biomass. Future research should address these issues by incorporating objective immune markers (e.g., immunoglobulins, frailty scores), using larger multi-center cohorts with participant-level clustered analyses, and normalizing EPS to viable cell counts or total biomass. Metabolomic and genomic characterization of L475 CFS and in vivo validation will also be essential to identify active compounds and assess antibiofilm potential.

Despite these constraints, this study provides a preliminary proof-of-concept investigation. It is among the few reports to link self-reported immune health in older adults to gut *E. coli* carriage, resistance, and biofilm formation, and it systematically identifies *L. paracasei* L475 as a potential candidate against a representative MDR isolate from this vulnerable population. The observed disruption of EPS and cell membranes indicates that *L. paracasei* L475 CFS has preliminary in vitro potential as a postbiotic against biofilm-associated MDR *E. coli*. However, this in vitro activity requires further investigation—including mechanistic controls and in vivo validation—before any practical application can be considered.

## Figures and Tables

**Table 1:** List of antibiotics tested and interpretive standards in this study.

CLSI Class	Antibiotic	Abbreviation	Concentration (μg/mL)	S * (mm)	I (mm)	R (mm)
Aminoglycosides	Amikacin	AK	30	≥20	17–19	≤16
	Gentamicin	GEN	10	≥18	15–17	≤14
	Streptomycin	S	10	≥15	12–14	≤11
Cephalosporins	Cefazolin	KZ	30	≥23	20–22	≤19
	Cefoxitin	FOX	30	≥18	15–17	≤14
	Ceftazidime	CAZ	40	≥21	18–20	≤17
Quinolones	Ciprofloxacin	CIP	5	≥26	22–25	≤21
	Nalidixic acid	NA	30	≥19	14–18	≤13
Penems	Meropenem	MEM	10	≥23	20–22	≤19
Penicillins	Ampicillin	AMP	10	≥17	14–16	≤13
Tetracyclines	Tetracycline	TE	30	≥15	12–14	≤11
Sulfonamides	Sulfamethoxazole-Trimethoprim	SXT	25	≥16	11–15	≤10
Phenicols	Chloramphenicol	C	30	≥18	13–17	≤10
β–lactam combination agents	Sulbactam-Ampicillin	SAM	20	≥15	12–14	≤11

* S: Sensitive; I: intermediate; R: resistance. The results were interpreted according to the Clinical and Laboratory Standards Institute (CLSI) M100, 33rd edition.

**Table 2:** Demographic characteristics of study subjects.

	Normal Immunity (H, N = 10)	Low Immunity (L, N = 10)	*p* Value
Sex			1.0
Female	5	5	
Male	5	5	
Age	63.70 ± 5.21	65.40 ± 5.02	0.467
BMI	24.51 ± 1.51	23.90 ± 1.68	0.404
Average number of colds/yr	1.30 ± 0.49	5.60 ± 0.66	<0.001
Cold recovery time/d	4.30 ± 1.27	8.90 ± 2.84	<0.001
Immunity self-assessment	8.20 ± 0.75	5.90 ± 0.54	<0.001

**Table 3:** Multidrug resistance among the *E. coli* isolates recovered from older individuals.

Antibiotic Classes with Resistance *	Resistance Phenotype	Number of MDR Strains		
		Total	H	L
Resistance to 3 classes				
Amin.-Quin.-Sulf.	S-NA-SXT	1	1	0
Ceph.-Quin.-Peni.	KZ-NA-AMP	1	0	1
Amin.-Quin.-Tetr.	S-NA-TE	2	0	2
Amin.-Quin.-Peni.	GEN-NA-AMP	1	1	0
	GEN/S-CIP/NA-AMP	1	0	1
Amin.-Ceph.-Quin.	GEN-FOX/CAZ-CIP	1	0	1
Resistance to 4 classes				
Amin.-Peni.-Sulf.-Phen.	GEN-AMP-SXT-C	2	2	0
Ceph.-Quin.-Peni.-Sulf.	KZ/CAZ-NA-AMP-SXT	1	1	0
Amin.-Quin.-Peni.-Tetr.	GEN/S-CIP/NA-AMP-TE	1	0	1
Amin.-Quin.-Peni.-β lac.	S-CIP/NA-AMP-SAM	1	0	1
Amin.-Ceph.-Quin.-Sulf.	GEN-FOX/CAZ-CIP-SXT	1	0	1
Resistance to 5 classes				
Amin.-Quin.-Peni.-Tetr.-Sulf.	S-NA-AMP-TE-SXT	1	0	1
	GEN/S-CIP/NA-AMP-TE-SXT	1	0	1
	S-CIP/NA-AMP-TE-SXT	3	0	3
Amin.-Quin.-Peni.-Sulf.-Phen.	GEN/S-CIP-AMP-SXT-C	1	1	0
Amin.-Quin.-Tetr.-Sulf.-Phen.	S-NA-TE-SXT-C	1	1	0
Amin.-Quin.-Peni.-Tetr.-β lac.	S-CIP/NA-AMP-TE-SAM	3	0	3
	S-NA-AMP-TE-SAM	2	0	2
	GEN/S-CIP/NA-AMP-TE-SAM	2	0	2
	S-CIP/NA-AMP-TE-SAM	1	0	1
Amin.-Quin.-Pene.-Tetr.-Sulf.	AK/S-NA-MEM-TE-SXT	1	0	1
Resistance to 6 classes				
Amin.-Ceph.-Quin.-Peni.-Sulf.-Phen.	S-KZ/CAZ-CIP/NA-AMP-SXT-C	4	0	4
	S-KZ/CAZ-CIP-AMP-SXT-C	1	0	1
Amin.-Ceph.-Quin.-Peni.-Tetr.-Sulf.	S-KZ/CAZ-CIP/NA-AMP-TE-SXT	1	0	1
Amin.-Quin.-Peni.-Tetr.-Sulf.-Phen.	GEN/S-CIP/NA-AMP-TE-SXT-C	5	1	4
Resistance to 7 classes				
Amin.-Ceph.-Quin.-Peni.-Tetr.-Sulf.-Phen.	S-KZ/CAZ-CIP/NA-AMP-TE-SXT-C	1	0	1
		41	8	33

* Antimicrobial classes: Aminoglycosides (Amin.): [Amikacin (AK), Gentamicin (GEN) and Streptomycin (S)]; cephalosporins (Ceph.) [Cefazolin (KZ), Cefoxitin (FOX) and Ceftazidime (CAZ)]; quinolones (Quin.) [Ciprofloxacin (CIP) and Nalidixic acid (NA)]; penems (Pene.) [Meropenem (MEM)]; pencillins (Peni.) [Ampicillin (AMP)]; tetracyclines (Tetr.) [Tetracycline (TE)]; sulfonamides (Sulf.) [Sulfamethoxazole-Trimethoprim (SXT)]; phenicols (Phen.) [Chloramphenicol (C)]; and β lactam combination agent (β lac.) [Sulbactam-Ampicillin (SAM)].

## Data Availability

The original contributions presented in this study are included in the article/Supplementary Material. Further inquiries can be directed to the corresponding authors.

## Supplementary Materials

The following supporting information can be downloaded at: https://www.mdpi.com/article/10.3390/microorganisms14040888/s1, Table S1: Number of *E.coli* isolates in total, selected and defined as MDR by participants. Figure S1: *Lactobacillus* tolerance to acidic conditions at pH 2.5 (a) and 0.5% bile salts (b). Figure S2: Viable cell counts of *E. coli* within biofilms after treatment with lactobacilli cell-free supernatants under (a) inhibition and (b) eradication conditions. Detection limit (0.7 log CFU/mL).

## Abbreviations

The following abbreviations are used in this manuscript:

MDR	Multidrug-resistant
CFSs	Lactobacillus cell-free supernatants
EPS	Extracellular polymeric substance
SEM	Scanning electron microscopy
CLSM	Confocal laser scanning microscopy
OD	Optical density
MBIC	Minimal biofilm inhibitory concentration
MBEC	Minimum biofilm eradication concentration
