# Supplementary material for: Antibiofilm Potential and Mechanisms of Lacticaseibacillus paracasei L475 Against Multidrug-Resistant Escherichia coli Isolated from Older Adults
Source: Microorganisms. 2026 Apr 16;14(4):888. doi: 10.3390/microorganisms14040888 (PMC13118906; doi:10.3390/microorganisms14040888)
Supplement: Supplementary file 1 [file microorganisms-14-00888-s001.zip › Figure S1.pdf]

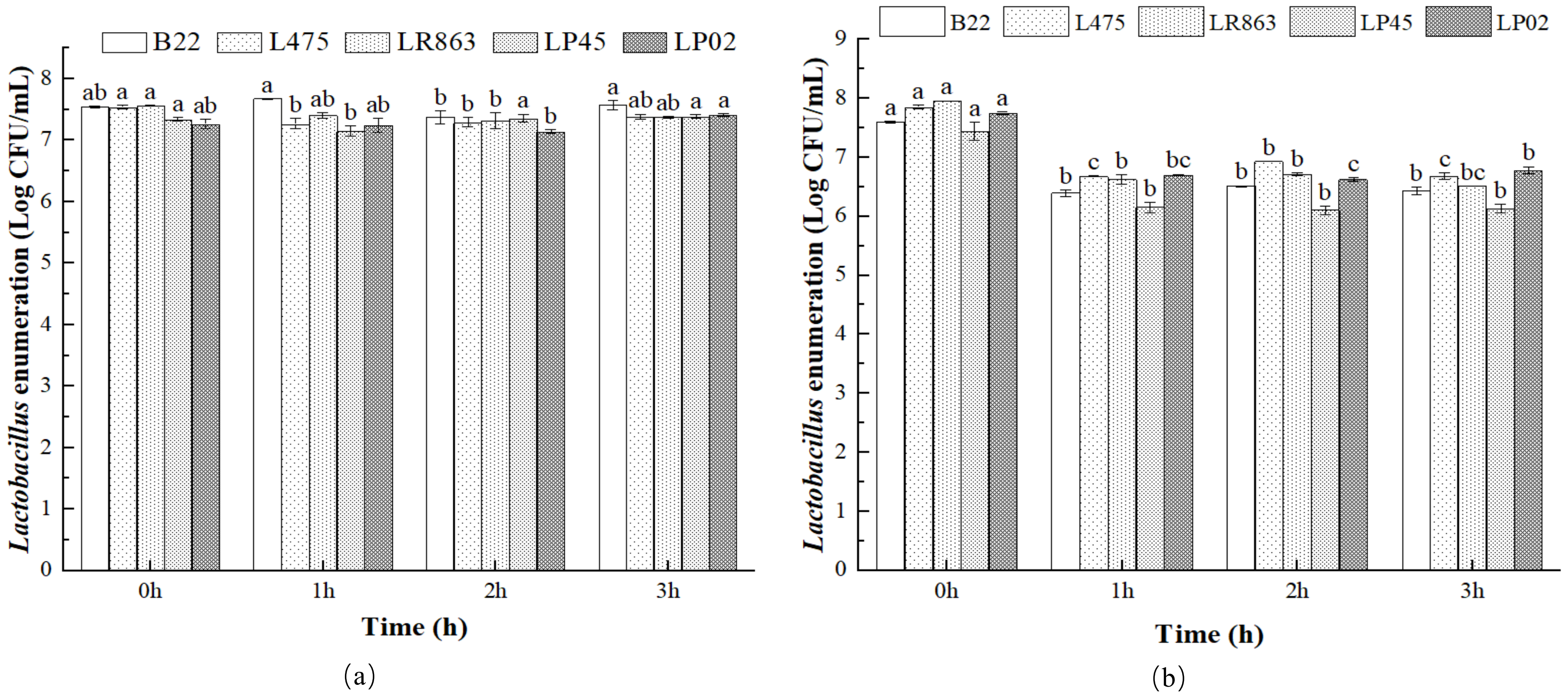

Figure .S1: *Lactobacillus* tolerance to acidic conditions at pH 2.5 (a) and 0.5% bile salts (b) . The strains B22, LP02, LP45, L475, and LR863 refer to *Lactobacillus plantarum* B22, *Lactobacillus paracasei* 02, *Lactobacillus plantarum* 45, *Lactobacillus paracasei* L475, and *Lactobacillus rhamnosus* 863, respectively. Different lowercase letters on the columns denote significant differences in the enumaration of same *Lactobacillus* strain at different treatment time points
