# Supplementary material for: Antibiofilm Potential and Mechanisms of Lacticaseibacillus paracasei L475 Against Multidrug-Resistant Escherichia coli Isolated from Older Adults
Source: Microorganisms. 2026 Apr 16;14(4):888. doi: 10.3390/microorganisms14040888 (PMC13118906; doi:10.3390/microorganisms14040888)
Supplement: Supplementary file 1 [file microorganisms-14-00888-s001.zip › Figure S2.pdf]

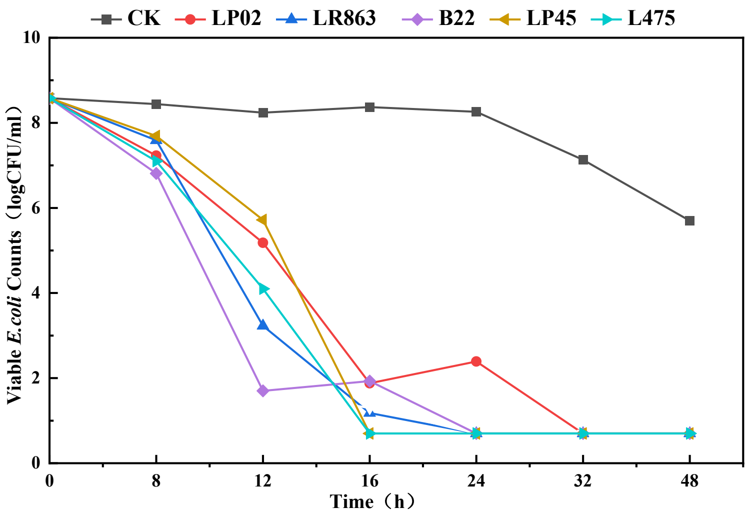

(a)

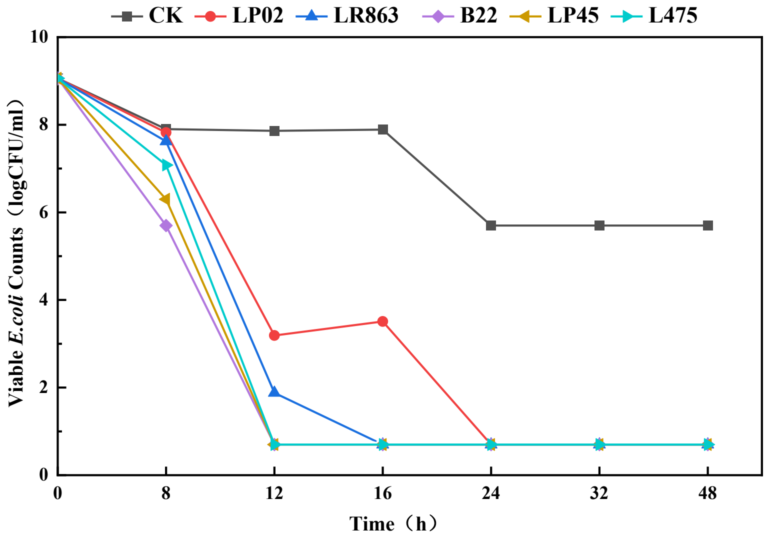

(b)

Fig. S2: Viable cell counts of *E. coli* within biofilms after treatment with lactobacilli cell-free supernatants under (a) inhibition and (b) eradication conditions. Detection limit (0.7 log CFU/ml)
