# Supplementary material for: Antibiofilm Potential and Mechanisms of Lacticaseibacillus paracasei L475 Against Multidrug-Resistant Escherichia coli Isolated from Older Adults
Source: Microorganisms. 2026 Apr 16;14(4):888. doi: 10.3390/microorganisms14040888 (PMC13118906; doi:10.3390/microorganisms14040888)
Supplement: Supplementary file 1 [file microorganisms-14-00888-s001.zip › Table S1.pdf]

Table S1 Number of *E.coli* isolates in total, selected and defined as MDR by participants.

Subjects	Group	No. of isolates in total	No. of isolate included	No. of MDR isolates
1	H	5	1	1
2	H	12	3	0
3	H	10	3	1
4	L	12	3	3
5	L	12	3	3
6	L	5	1	0
7	H	5	1	0
8	H	5	1	0
9	L	12	3	2
10	H	8	2	0
11	H	16	5	0
12	L	14	4	4
13	H	8	2	2
14	L	8	3	2
15	H	25	9	2
16	H	16	6	2
17	L	15	6	6
18	L	9	3	3
19	L	10	3	3
20	L	34	8	7
Sum		241	70	41
